# Genetic Variations in TERT-CLPTM1L Genes and Risk of Lung Cancer in Chinese Women Nonsmokers

**DOI:** 10.1371/journal.pone.0064988

**Published:** 2013-05-30

**Authors:** Cheng Li, Zhihua Yin, Wei Wu, Xuelian Li, Yangwu Ren, Baosen Zhou

**Affiliations:** 1 Department of Epidemiology, School of Public Health, China Medical University, Shenyang, China; 2 Key Laboratory of Cancer Etiology and Intervention, University of Liaoning Province, China; University of Birmingham, United Kingdom

## Abstract

**Background:**

The TERT gene is the reverse transcriptase component of telomerase and is essential for the maintenance of telomere DNA length, chromosomal stability and cellular immortality. CLPTM1L gene encodes a protein linked to cisplatin resistance, and it is well conserved and express in various normal or malignant tissues, including lung.

**Methods:**

To test this hypothesis, we genotyped for two significant SNPs TERT-rs2736098 and CLPTM1L-rs4016981 in a case-control study with 501 cancer cases and 576 cancer-free controls in Chinese nonsmoking population. Information concerning demographic and risk factors was obtained for each case and control by a trained interviewer. Gene polymorphisms were determined by TaqMan methodology.

**Results:**

We found that the homozygous variant genetic model of TERT gene was associated with a significantly increased risk of lung cancer with adjusted OR of 1.72(95%CI = 1.19–2.51, *P* = 0.004 for heterogeneity). The joint effect of TERT and CLPTM1L increased risk for lung cancer with adjusted OR is 1.31(95%CI = 1.00–1.74, *P* = 0.052 for heterogeneity).

**Conclusion:**

Genetic variants in TERT and CLPTM1L may affect the susceptibility of lung cancer, especially adenocarcinoma in Chinese women nonsmokers.

## Background

Worldwide, cancer is one of the most leading cause of cancer-related deaths. Most types of cancer are attributed to gene-environment interaction effect. A large number of surveys have focused on gene polymorphism and the cancer susceptibility. In addition to several types of inherited tumor, most sporadic cases is still undefined. The latest studies pay attention to telomerase reverse transcriptase(TERT), cleft lip and palate transmembrane 1-like(CLPTM1L) gene variation which locus at chromosome 5p15.33. Many genome-wide association studies(GWAS) of cancer have been reported and provided that the TERT-CLPTM1L genomic region associated the risk of lung cancer. In most of studies, the main of cases and controls were cigarette smokers that made it difficult to judge whether these genes were associated with lung cancer or tobacco use [1]. Furthermore, a substantial proportion of lung cancer in Asian women occurs among non-smokers, who interestingly have a relatively high rage of cancer [2]. Moreover, previous study showed the evidence that associations of genotype polymorphisms with lung cancer risk could be confounded by gender or smoking [3]. Therefore, we conducted a genome-wide association study in never-smoking females in Chinese Han population.

The TERT gene is the reverse transcriptase component of telomerase and is essential for the maintenance of telomere DNA length, chromosomal stability and cellular immortality [4], [5]. Telomerase activity was found in 85%–90% of human cancers. Furthermore, telomere length in most tumors would be maintained for the sake of the abnormally express of the telomere in malignant cells such as most of tumor cells. However this phenomenon have not revealed in health tissue or cells [6]. CLPTM1L gene encodes a protein linked to cisplatin resistance ant it is well conserved and express in various normal or malignant tissues, such as skin lung, breast, ovarian cervical [7].

Previous studies reported that TERT gene and CLPTM1L gene associated to the risk of many type of cancers. Recently, two genome-wide association studies have identified the same susceptible region for lung cancer. Among these sites, rs2736098 which locus in TERT gene has been identified the susceptible. Simultaneously, rs401681 are in a region of high linkage disequilibrium (LD) that includes the promoter regions of TERT and the entire coding region of the CLPTM1L gene also reported a lot. To test this hypothesis, we genotyped for two significant SNPs TERT-rs2736098 and CLPTM1L-rs4016981 in a case-control study with 501 cancer cases and 576 cancer-free controls in Chinese non-smoking population.

## Materials and Methods

### Study Subject

We obtained ethics approval for our study. The name of the ethics committee is China Medical University Ethics Committee. We also upload a formal written waiver of ethics approval to your system.

This was a hospital-based case-control study in the northeast of China. All subjects were unrelated ethnic Han Chinese. There were no restriction of age and histology for the recruitment and the exclusion criteria of the cases included metastasized cancer, previous cancer and previous radiotherapy or chemotherapy. A total of 501 lung cancers cases were obtained from 2002 to 2010 at the first affiliated Hospital of China Medical University and Liaoning cancer histopathologically confirmed primary lung cancer. The 576 cancer-free controls were selected from the same hospital or present history of malignant disease during the same time period as the cases were recruited and they were frequency matched to case subjects by age (±5 years). Controls suffered mainly from bronchitis, pulmonary disease and emphysema. All individuals included in this study were only enrolled after providing written informed consent to participate.

### Genotype Analysis

Genomic DNA was extracted from a leukocyte pellet using proteinase K digestion followed by phenol-chloroform extraction and ethanol precipitation as described previously [8]. SNPs in both TERT-rs2736098 and CLPTM1L-rs401681 were genotyped by investigators blinded to case-control status in order to avoid any genotyping bias, using TaqMan methodology and read with the Sequence Detection Software on an Applied Bio-systems 7500 FAST Real-Time PCR System according to the manufacturer’s instructions (Applied Biosystems, Foster City, CA). PCR TaqMan primers and probes were supplied by Applied Biosystems. Each plate included one negative control (no DNA), duplicated commercial positive controls (TaqMan Control Genomic DNA; Applied Biosystems). Amplification was done under the following conditions: 95°C for 10 min followed by 47 cycles of 92°C for 30s and 60°C for 1 min. To validate the genotyping results, 10% randomly selected samples were duplicated and all duplicated were matched.

### Statistical Analysis

Two-sided *x* ^2^ test was used to examine differences in the distributions of demographic characteristics, selected variables and genotypes among cases and controls. The associations of genotypes of TERT and CLPTM1L with risk of lung cancer were estimated by computing the odds ratios (ORs) and Their 95% confidence interval (CIs) from both univariate and multivariate logistic regression models in case-control analysis. The Hardy-Weinberg equilibrium for each SNP was tested with Pearson’s *x* ^2^ test. All the analyses were performed with adjustment for age and income and all the individuals were non-smoking women. All tests were two sided, and P<0.05 was considered significant. All analyses were performed using the Statistical Product and Service Solutions software (v.16.0; SPSS Institute Cary, Chicago, IL).

## Results

The demographic data for 501 lung cancer cases and 576 cancer-free controls are presented in Table 1, with mean ages of cases and controls (mean ±S.D.) being almost identical (56.7±11.6 and 56.2±13.0 years, respectively ). There was no significant difference on age between the cases and controls (*P* = 0.471). Cases included 334 adenocarcinomas, 90 squamous cell carcinomas and 77 other tumors with a variety of different pathologies (including large-cell, small-cell carcinomas and mixed cell or undifferentiated carcinomas). All the individuals were non-smoking females.

**Table 1:** Characteristics of lung cancer cancers and controls.

Characteristics	Cases	Controls	*P* value^a^
	(n = 501)	(n = 576)	
Female	501	576	
Mean age (±SD)	56.7±11.6	56.2±13.0	0.471
Histological type			
Adencarcinoma	334		
Squamous cell carcinoma	90		
Other	77		
smoking status			
never smoker	501	576	

a Two-sided *x* ^2^ test.

The observed genotype frequencies among the control subjects were both in agreement with that expected under the Hardy-Weinberg equilibrium (p^2^+2pq+q^2^ = 1, *P* = 0.065 for rs2736098 and 0.463 for rs401681). As shown in Table 2, the rs2736098 T allele in TERT gene was associated with a significantly increased risk of lung cancer (*P* = 0.009 for heterogeneity), with adjusted ORs of 1.27(95%CI = 1.06–1.52). Meanwhile, we could observe an increased association between the rs2736098 polymorphism and the risk of lung cancer in one genetic model (TT vs CC: OR = 1.72, 95%CI = 1.19–2.51, *P* = 0.004 for heterogeneity). In addition, when we combined the variant genotypes (TT+TC) assuming a dominant genetic model, the combined variant genotypes non-significantly increased the susceptibility of lung cancer. However, no significant association was showed among each genetic model between rs401681 in CLPTM1L and lung cancer risk. Then we combined the two SNPs to evaluate their joint effect on risk of lung cancer. We categorized all genotypes of each SNP into the three or two different groups (trichotomized and dichotomized) according to the number of variant genotypes. As shown in Table 2, for dichotomized groups, individuals with 2 variant genotypes had a significantly increased risk for lung cancer, compared with individuals with 0 or 1 variant genotypes. The adjusted OR is 1.31(95%CI = 1.00–1.74, *P* = 0.052 for heterogeneity).

**Table 2:** Genotype frequencies of the TERT and CLPTM1L polymorphisms among lung cancer cases and controls and their associations with risk of lung cancer.

Genotypes	NO(%)		*P* value	Adjusted OR(95%CI)^b^
	Cases^a^	Controls^a^		
TERT-rs2736098				
CC	173(37.0)	227(41.7)		1.00
CT	207(44.2)	250(46.0)	0.548	1.09(0.82–1.42)
TT	88(18.8)	67(12.3)	0.004	1.72(1.19–2.51)
CT+TT	295(63.0)	317(58.3)	0.123	1.22(0.95–1.57)
T alleles^c^			0.009	1.27(1.06–1.52)
CLPTM1L-rs401681				
TT	41(8.8)	58(10.8)		1.00
CT	205(44.2)	234(43.7)	1.264	1.26(0.81–1.96)
CC	218(47.0)	244(45.5)	1.239	1.24(0.80–1.93)
CT+CC	423(91.2)	478(89.2)	0.295	1.25(0.82–1.91)
C alleles			0.401	1.08(0.90–1.31)
Trichotmomize combined^d^				
0	68(16.7)	82(17.4)		1.00
1	76(18.6)	114(24.3)	0.323	0.80(0.52–1.24)
2	264(64.7)	274(58.3)	0.418	1.16(0.81–1.67)
Dichotomize combined^d^				
0–1	144(35.3)	196(41.7)		1.00
2	264(64.7)	274(58.3)	0.052	1.31(1.00–1.74)

a All the individuals were non-smoking women.

b Adjusted by age.

c Allelic ORs.

d 0 group, TERT CC genotype and CLPTM1L TT genotype;1 group, TERT CC genotype and CLPTM1L variant genotypes or CLPTM1L TT genotype and TERT variant genotypes; 2 group, TERT variant genotypes and CLPTM1L variant genotypes.

Table 3 summarized the relationship between the TERT-rs2736098 and CLPTM1L-rs401681 genotypes in lung cancer cases and controls, stratified by histology type. The rs2736098 T allele in TERT gene was associated with a significantly increased risk of lung cancer in adenocarcinoma subgroup (P<0.001 for heterogeneity), with adjusted ORs of 1.43(95%CI = 1.17–1.75). We also obtained a statistically significant increased association between the rs2736098 polymorphism and adencarcinoma risk in two genetic models (TT vs CC: OR = 2.17, 95%CI = 1.44–3.25, *P*<0.001 for heterogeneity; CT+CC vs CC: OR = 1.40, 95%CI = 1.05–1.86, *P* = 0.026 for heterogeneity). Also as described in Table 3, C allele in CLPTM1L-rs401681 increased the risk of lung adenocarcinoma subgroup and the adjusted OR is 1.23(95%CI = 1.00–1.52, *P* = 0.046 for heterogeneity).

**Table 3:** The effect of the SNPs rs2736098 and rs401681 in the region on lung cancer risk in pathological subgroup.

Genotypes	NO(%)		*P* value	Adjusted OR(95%CI)^b^
	Cases	Controls		
TERT rs2736098				
AC^a^				
CC	108(34.0)	227(41.7)		1.00
CT	140(44.2)	250(46.0)	0.269	1.19(0.87–1.62)
TT	69(21.8)	67(12.3)	<0.001	2.17(1.44–3.25)
CT+TT	209(66.0)	317(58.3)	0.026	1.40(1.05–1.86)
T alleles			<0.001	1.43(1.17–1.75)
SCC^b^				
CC	35(43.8)	227(41.7)		1.00
CT	35(43.8)	250(46.0)	0.908	0.91(0.55–1.50)
TT	10(12.4)	67(12.3)	0.968	0.97(0.46–2.06)
CT+TT	45(56.2)	317(58.3)	0.732	0.92(0.57–1.48)
T alleles			0.820	0.96(0.68–1.36)
CLPTM1L rs401681				
AC				
TT	32(10.2)	58(10.8)		1.00
CT	137(43.5)	234(43.7)	0.739	1.09(0.67–1.75)
CC	146(46.3)	244(45.5)	0.772	1.07(0.66–1.74)
CT+CC	283(89.8)	478(89.2)	0.743	0.93(0.59–1.46)
C alleles			0.046	1.23(1.00–1.52)
SCC				
TT	6(8.5)	58(10.8)		1.00
CT	19(26.8)	234(43.7)	0.301	1.56(0.67–3.64)
CC	46(64.7)	244(45.5)	0.884	1.07(0.47–2.55)
CT+CC	65(91.5)	478(89.2)	0.507	0.76(0.33–1.72)
C alleles			0.086	1.34(0.96–1.87)

a Adenocarcinomas.

b Squamous cell carcinomas.

## Discussion

To our knowledge, this is the first study that has investigated whether the two GWAS-identified genetic variants (TERT-rs2736098 and CLPTM1L-rs401681) at the 5p5.33 locus are associated with lung cancer risk in never-smoking Han-Chinese females. The results suggested that the variant allele gene of rs2736098 was significantly associated with increased risk of lung cancer, especially in lung adenocarcinoma. On the other side, our study obtained the data about the increased risk of lung adenocarcinoma and the rs401681 C allele in CLPTM1L gene, whereas the other genetic models had no statistically significant association with lung cancer in women nonsmokers.

The TERT gene has been mapped to chromosome 5p15.33 and consisted of 16 exons and 15 introns spanning 35kb of genomic DNA [9], rs2736098 is localized to the second exon of the telomerase gene TERT. TERT protein is a catalytic subunit and a key regulator of telomerase activity. Therefore, TERT was considered to be a more plausible candidate at 5p15.33 for lung cancer risk [4]. Overexpression of TERT and telomerase activity has been observed in many tumors, such as lung cancer, skin cancer, glioma. Nevertheless, the functional significance of the SNP rs2736098 was not clear and the molecular mechanism by which the polymorphism affects lung cancer risk is not yet elucidated. Our research provided the strong evidence from Chinese population that the 5p15.33 might be causal region to predispose to lung cancer susceptibility, and the effect of the SNP rs2736098 was the same to the Korean population in Asia [10]. So far, none of the studies concerned the relationship between rs2736098 in TERT and lung cancer in Caucasian population, although we found that the mutation allele frequency in Caucasian population was 37% through HapMap as well as the data in Asian population was 41% (http://snp.cshl.org/cgi-perl/gbrowse/hapmap24_B36/). The CLPTM1L gene also located on chromosome 5p15.33 and it may play a role in apoptotic response. In addition, CLPTM1L is also an attractive cancer susceptibility gene as it encodes a transcript whose overexpression has been linked to cisplatinum resistance [7]. Overexpression of CLPTM1L mRNA has been observed in many cancer types, including lung cancer. Base on our results, rs401681 variant allele of TERT not contribute to lung cancer risk for women nonsmoker. Nonetheless, when stratified by pathology, the C allele in CLPTM1L-rs401681 increased the risk of lung adenocarcinoma subgroup and the adjusted OR is 1.23(95%CI = 1.00–1.52, *P* = 0.046 for heterogeneity). Therefore, our study suggested that CLPTM1L-rs401681 variant genotypes may play different roles in different pathology subgroups of lung cancer. Looking back on previous studies, the results that have been reported were unsatisfactory and even conflicting. For example, an early study reported that the CLPTM1L T genotypes were not associated with risk of lung cancer in a Caucasian population [11]. Moreover, two researches reported that CLPTM1L gene polymorphism was not associated with risk of lung cancer in an Asian population [15], [16]. Both of them were hospital-based data sets. Nevertheless, eight association studies showed that rs401681 allele C marginally increased overall lung cancers risk both in a Caucasian and an Asian population[3], [12], [17]–[22]. Not long ago, a meta-analysis researched by Yin et al. found that individuals with the C allele genotypes had higher risk of lung cancer [23]. Though that study, strikingly increased risk was found in smoking related cancers, such as lung cancer. It may be explained that CLPTM1L protein may be involved in the apoptosis response of genotoxic stress. The possible explanation for these discrepancies could be that the allele frequencies in different ethnicity were quite different. Data in HapMap demonstrate that the T allele frequency in Caucasian population was 43%, but the data in Asian population was 29% (http://snp.cshl.org/cgi-perl/gbrowse/hapmap24_B36/). The distinct also could because of the great difference in each sample sizes. Most of the latest GWAS studies contained more that ten thousand cases as well as controls. Conversely, when using TaqMan or PCR-RFLP methodology, the other researches contained almost one thousand cases or just several hundred cancer cases. Moreover, the discrepancies could due to different source of control group or genotyping method. The exact functional of CLPTM1L remains unclear. The previous study speculated it may be in strong LD with other potential functional or causal SNPs [7]. Further more, a positive relationship between rs401681 C allele and shorter relative telomere length suggested that CLPTM1L gene was a candidate in affecting lung cancer susceptibility [24]. It was same to the TERT-rs2736098. In addition, base on the potential interact effect between TERT and CLPTM1L, we categorized all genotypes of each SNP into two different groups according to the number of variant genotypes. The dichotomize model indicated that the individuals with 2 variant genotypes had a significantly increased risk for lung cancer, compared with individuals with 0 or 1 variant genotypes.

TERT-CLPTM1L genomic region have been extensively studies in many cancer sites in different populations, but the results are conflicting. Gago-Dominguez et al. [13] found that variant allele gene in both TERT-rs2736098 and CLPTM1L-rs401681 were significantly associated with increased risk of bladder cancer among Chinese population. Nevertheless, there was lack of association between common single nucleotide polymorphisms in the TERT-CLPTM1L locus and breast cancer in non-Hispanic whites. Also Chen et al. [14] reported that homozygous variant genetic model of TERT-rs2736098 increased risk of glioma. It is noteworthy that TERT-CLPTM1L genomic region among Asian population has been linked to increased risk of several cancer types. Conversely, lack of studies researched other ethnicities. More evidences should be provided in future regarding the association between TERT-CLPTM1L genomic region and cancer risk in different races. Further studies that take into consideration subgroup analysis and environmental stresses in this region are necessary.

### Conclusion

In conclusion, our study revealed that TERT-rs2736098 polymorphism may increase the risk of lung cancer, especially in lung adenocarcinoma in women nonsmokers in Chinese population. Moreover, C allele in CLPTM1L-rs401680 gene may alter the risk of lung adenocarcinoma. Because genetic polymorphisms often vary between ethnic groups, this investigation could be extended in well-designed future studies in diverse ethnic women nonsmokers.
